# Velocity distributions in trapped and mobilized non-wetting phase ganglia in porous media

**DOI:** 10.1038/s41598-018-31639-4

**Published:** 2018-09-05

**Authors:** I. Zarikos, A. Terzis, S. M. Hassanizadeh, B. Weigand

**Affiliations:** 1 Environmental Hydrogeology Group, Dept. of Earth Sciences, University of Utrecht, 3584 CD Utrecht, The Netherlands; 2 Institute of Aerospace Thermodynamics, University of Stuttgart, 70569 Stuttgart, Germany

## Abstract

Understanding the mobilisation of trapped globules of non-wetting phase during two-phase flow has been the aim of numerous studies. However, the driving forces for the mobilisation of the trapped phases are still not well understood. Also, there is little information about what happens within a globule before, at the onset and during mobilization. In this work, we used micro-particle tracking velocimetry in a micro-fluidic model in order to visualise the velocity distributions inside the trapped phase globules prior and during mobilisation. Therefore, time-averaged and instantaneous velocity vectors have been determined using fluorescent microscopy. As a porous medium, we used a polydimethylsiloxane (PDMS) micro-model with a well-defined pore structure, where drainage and imbibition experiments were conducted. Three different geometries of trapped non-wetting globules, namely droplets, blobs and ganglia were investigated. We observed internal circulations inside the trapped phase globules, leading to the formation of vortices. The direction of circulating flow within a globule is dictated by the drag force exerted on it by the flowing wetting phase. This is illustrated by calculating and analyzing the drag force (per unit area) along fluid-fluid interfaces. In the case of droplets and blobs, only one vortex is formed. The flow field within a ganglion is much more complex and more vortices can be formed. The circulation velocities are largest at the fluid-fluid interfaces, along which the wetting phase flows and decreases towards the middle of the globule. The circulation velocities increased proportionally with the increase of wetting phase average velocity (or capillary number). The vortices remain stable as long as the globules are trapped, start to change at the onset of mobilization and disappear during the movement of globules. They reappear when the globules get stranded. Droplets are less prone to mobilization; blobs get mobilised in whole; while ganglia may get ruptured and get mobilised only partially.

## Introduction

Immiscible two-phase flow in porous media is encountered in a large variety of applications and industrial processes. In some cases, such as in oil and gas industry as well as in soil remediation, one of the liquid phases (oil, gas, or organic liquid pollutants, which are the nonwetting phase) remains partially trapped and cannot be recovered. In some other cases, such as in CO_2_ sequestration, the aim is the trapping of CO_2_ in the porous medium, ensuring its permanent storage. While these applications have different goals, they all share the presence of a fluid phase that does not occupy a contiguous space. The major challenge with the presence of a discontinuous phase during two-phase flow is the correct macroscopic description of the flow of both continuous and trapped phases. Traditional macroscopic two-phase flow theories are based on Darcy’s law, which is valid only if both fluid phases occupy contiguous domains. Also, for developing a physically-based theory for the description of movement of a discontinuous phase, the underlying physical processes and mechanism must be unravelled. In particular, the momentum transfer between the two fluid phases and the role of the flow of continuous phase in the movement of the discontinuous phase is of major importance. Therefore, the experimental investigation of the behaviour of the trapped phase and its mobilization, are of major importance for the understanding of its behaviour and consequently the formulation of a macroscopic theory.

When a porous medium, initially saturated with a non-wetting phase, is flooded with a wetting phase, even at relatively high saturations of the non-wetting phase, some of it may break into dispersed clusters. Theses clusters are named ganglia or bubbles, in the case of liquid or gaseous phases, respectively. More specifically, dispersed clusters of the nonwetting phase are classified into three categories based on their size and shape: droplets, blobs and ganglia. The smallest body of trapped non-wetting phase is called droplet; these are relatively round bodies that fit within a single pore and are surrounded by the wetting phase. Larger globules are called blobs; they occupy a single pore fully. Finally, larger clusters of non-wetting phase, which occupy more than one pores, are referred to as ganglia. Payatakes *et al*.^1^ defined ganglion as a nodular blob of a non-wetting phase that occupies at least one and usually several adjoining chambers of the void space.

**Figure 1:**
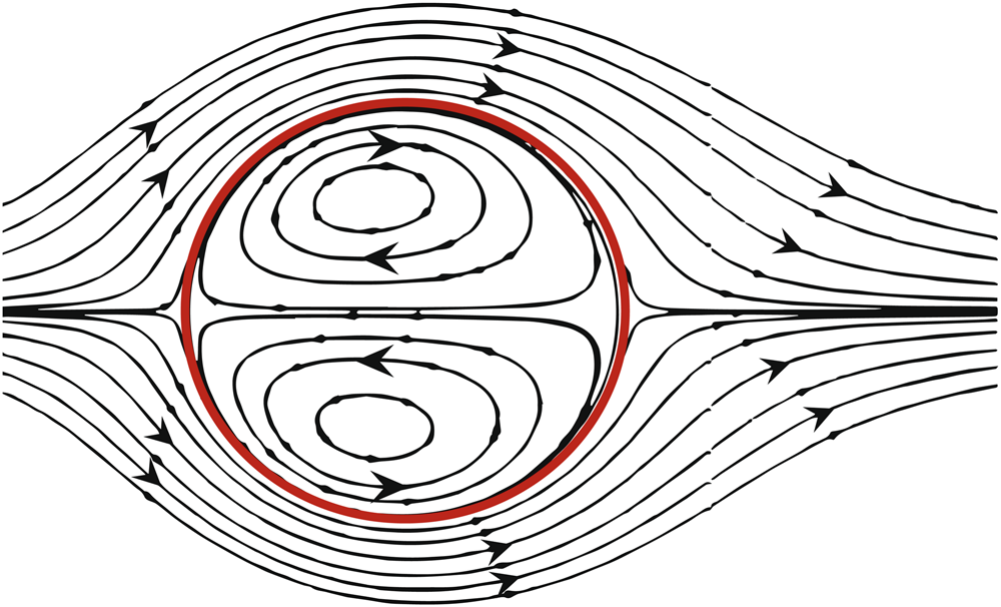
Internal circulation in the form of counter-rotating vortices. The red circle represents the liquid–liquid interface between the liquid sphere and the fluid flowing around it. Adopted and modified from Dong & Sau^11^.

Trapping and mobilisation of trapped non-wetting phase has been the subject of numerous studies. In these studies, the behaviour of trapped phase has been linked to a variety of parameters, such as capillary number, viscosity ratio and fluid topology. Here, we define the capillary number as the ratio of product of average wetting phase flow velocity and its viscosity to the fluid-fluid interfacial tension. It is observed that the mobilisation or complete removal of ganglia from the host porous medium is possible only if the wetting phase flows at high capillary numbers^2–8^. The effect of capillary number, Ca (usually defined in terms of the wetting phase), on trapping and mobilization of ganglia has been extensively studied^1,4,5,9^. Experiments have shown that the increase of Ca leads to a bigger chance of ganglia mobilisation, regardless of other physical and flow parameters^10^. It is reported that for the mobilization of the trapped non-wetting phase to occur, the imposed capillary number must be 25 times larger than the capillary number at which trapping occurred^4^. In the case of sandstone, in order to maximize recovery of the trapped non-wetting phase, the applied Ca number should be 100 times larger than the capillary number required for trapping^5^. Figure 1 shows the internal circulation in the form of counter-rotating vortices within a stationary droplet surrounded by another flowing fluid, marked by a red circle, as adopted and modified from Dong & Sau^11^.

The viscosity ratio has also a direct effect on the flow regime and the trapping of non-wetting phase^12^. In particular, it affects the size of ganglia formed during imbibition. Unfavourable viscosity ratio conditions during drainage, i.e. when the non-wetting phase viscosity is larger than that of the wetting phase, is known to cause capillary fingering, which in turn may lead to the formation of large ganglia. In cases of favourable viscosity ratio, however, the ganglia sizes are significantly smaller, since the advancing front is macroscopically smooth^13^. As a result, the extent of viscous fingering affects the size of the ganglia^14^. The importance of viscosity ratio is even greater on the mobilization of trapped ganglia and the reduction of residual saturation. For favourable viscosity ratios, the increase of Ca leads to a larger decrease of residual saturation. For unfavourable conditions, the residual saturation increases up to a Ca threshold value before it decreases^13^. Viscosity ratio has a distinct effect on the average velocity of ganglia too. Under favourable viscosity ratio conditions, the velocity of ganglia is similar or comparable with that of the continuous phase. For unfavourable conditions, however, the velocity is smaller. In addition to these observations, under favourable viscosity ratio conditions, droplets, blobs and ganglia occupying one pore, tend to move with larger velocities than larger ganglia^14,15^. This effect has not been observed under unfavourable conditions.

The initial distribution of the trapped phase is known to play a major role in the mobilisation potential of the phase^12,16–24^. In experimental works by Schlüter *et al*.^25^, it has been observed that high initial connectivity of trapped non-wetting phase clusters and the orientation along the flow direction lead to the mobilization of the ganglia and thus a lower residual non-wetting phase saturation. The majority, 65–90%, of the residual saturation has been observed to be linked to the population of large ganglia^20,22^, while small ganglia have a minor contribution to residual saturation. Although large ganglia have a distinct effect on residual non-wetting phase saturation, their population is significantly smaller than that of medium and small ganglia. The ganglion size plays an important role on their mobilisation^12^. Some have reported that larger ganglia or elongated ganglia are prone to easier mobilization. This is due to the local pressure difference on the two sides of a ganglion^20,22,26^. However, Morrow^21^ stated that elongated ganglia tend to resist mobilization. Others have also reported that shorter ganglia tend to have higher transport rates than larger ones^2–8^. The main mechanisms for mobilization of clusters are the break-up and coalescence of the ganglia, which occurs only to the large ganglia^16^.

Despite the wealth of literature focused on the entrapment and mobilisation of ganglia, there is no experimental study of the momentum exchange between two fluid phases and its effect on the remobilisation and movement of the non-wetting phase. There are some two-phase systems, albeit not in porous media, where such studies have been carried out. One example is the flow around droplets inside another fluid^27–31^. In case of spherical droplets, an internal circulation in the form of counter-rotating vortices, shown in Fig. 1, has been observed^11,32,33^. This is due to the momentum transfer between the two immiscible fluids, which depends strongly on their viscosity ratio^34,35^. Similar recirculations have been observed within falling droplets, with their intensity influenced by the resulted drag coefficient^36^. Another situation analogous to the ganglia in porous media is the movement of microfluidic droplets in micro-channels^37–41^. Experimental and numerical works on liquid-liquid slug flows in capillary channels have shown internal recirculations within both phases, which were affected by capillary forces^42^, the viscosity ratio between the fluids^43,44^, the channel flow velocity and the slug size^45,46^. Low capillary numbers resulted in slower channel flows and large slugs caused the attenuation of recirculation zones inside the mobilised slugs. There are two fundamental differences between such two-phase flow systems and the case of entrapped non-wetting phases in porous media. One is the presence of capillary forces that can cause the trapping of ganglia and the other is the blocking of droplets or ganglia by the solid phase. The geometry of the porous medium is of course much more complex than a micro-channel and large local variations in wetting phase velocity field may exist. Stagnant areas may exist where the wetting phase velocity could be close to zero. In such areas, the viscous drag exerted on the fluid-fluid interface would be negligible and thus there would no momentum exchange between the two fluid phases. These are passive interfaces. In areas that the wetting phase velocity is nonzero, we have active interfaces, where momentum exchange occurs, resulting in circulations within the ganglia.

The mobilization of non-wetting phase globules is controlled by the following four forces: drag forces exerted by the flowing wetting phase, friction exerted by the solid phase, capillary forces and the pressure differences in the wetting phase across a globule. As explained above, the drag force is believed to cause flow circulation within the globule. In fact, in order to quantify the drag force, one needs to measure detailed velocity field in the vicinity of the fluid-fluid interface within either the wetting phase or the non-wetting phase. In other words, knowledge of induced flow inside trapped non-wetting globules is important for the overall understanding of their fate. An effective technique for obtaining such information is microscopic particle velocimetry, where florescent tracers within the fluids are imaged in order to obtain the fluid velocity field. There are two major approaches: Particle Tracking Velocimetry (PTV) and Particle Imaging Velocimetry (PIV). PTV is a Lagrangian-based approach and provides trajectories and velocity magnitudes of flowing fluid, where PIV is Eulerian based and gives the velocity contour lines. Reviews of these techniques can be found in the work of Lindken *et al*.^47^. These techniques have been widely used in microfluidic devices^33,36,38,39^. x In this study, we have developed a micromodel setup and employed microscopic particle tracking velocimetry (*μPTV*) in order to visualise and quantify the flow inside trapped non-wetting phase. Our aim is to follow the development of velocity field within individual droplets, blobs and ganglia as the wetting phase capillary number increases, until the mobilisation of trapped phase occurs. We quantify the drag force exerted along the fluid-fluid interfaces and discuss its role in determining the direction of circulating flow within a globule. We discuss the reasons behind the fact that some non-wetting phase globules mobilise and some do not. Our highly-controlled experiment and detailed information about the velocity field within the trapped phase elements and mobilization of some of them will be also valuable for the testing and validation of direct pore-scale simulation methods such as Lattice-Boltzmann models.

## Materials and Methods

**Figure 2:**
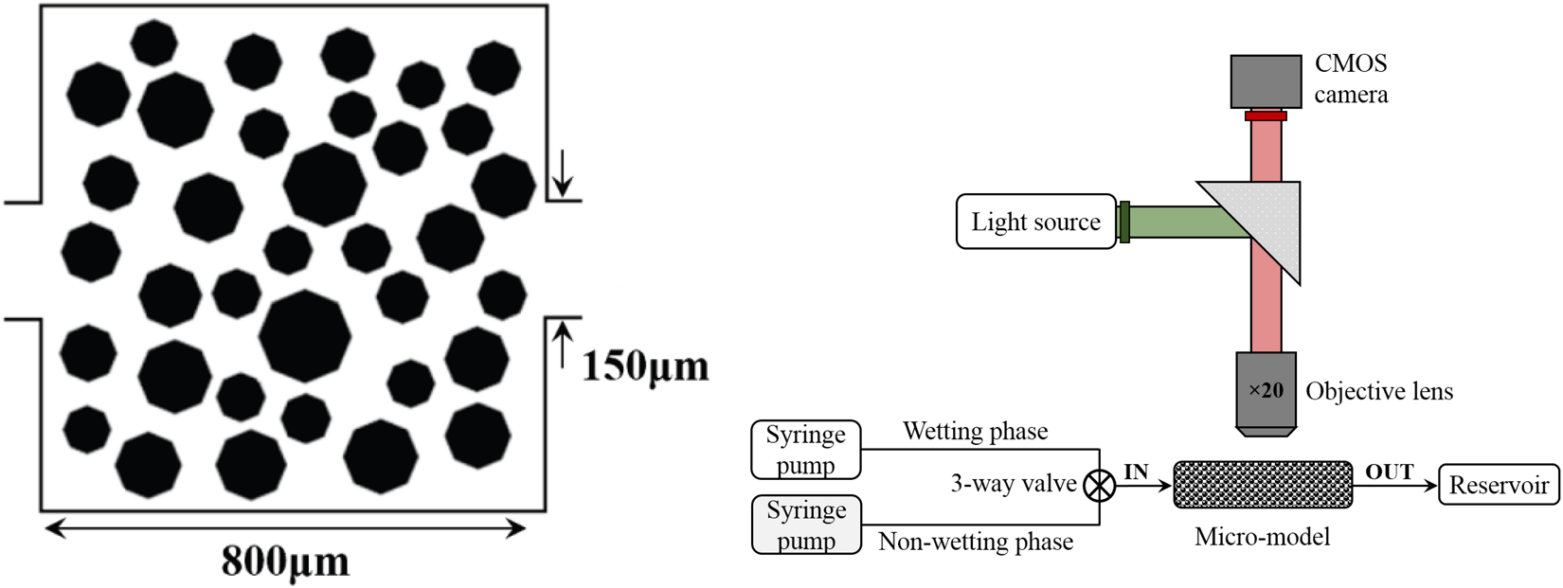
Geometrical characteristics of the microfluidic model (left) and schematic representation of the setup (right). The micro-model is square and has a depth of 50 *μm*.

The experiments were conducted in a porous medium represented by a square 2D micro-model. The micro-model was made of polydimethylsiloxane (PDMS) and its fabrication was based on a standard soft lithography technique, described in detail by Zarikos *et al*.^48^ and Karadimitriou *et al*.^49^, among others. The porous medium was generated by a random distribution of 35 octagonal pillars in a square domain of 800 × 800 (*μm*^2^). The pillars had a normal size distribution with a mean radius of 54 *μm* and minimum and maximum values of 46 *μm* and 89 *μm*, respectively. A schematic representation of the micro-model, with the solid pillars in black, is shown in Fig. 2(a). The thickness (or depth) of the micro-model was 50 *μm*, which was close to the average pore throat size. This means that the pores have an aspect ratio close to 1:1 and that capillarity phenomena are not dominated by the micro-model depth. The inlet of the porous network was connected through a 3-way valve with two long tubes for the injection of the liquid phases while the outlet tube was connected to the drain reservoir, as shown in Fig. 2(b). Either side of the micro-model could be used as inlet; with the other side becoming the outlet.

We used a low surface tension electronic liquid (Fluorinert FC-43, 15.8 mN/m) as the wetting phase and distilled water as the non-wetting phase. Water was doped with a fluorescent tracer. This was made of spherical Rhodamine 6G particles with a diameter of 0.86 *μm* and density of 1.05 *kg*/*m*^3^ density. The micro-model was initially saturated with the wetting phase (Fluorinert). Then it was drained by injecting the non-wetting phase (doped water). After that, the main imbibition was initiated, with the injection of the wetting phase, leading to the disconnection of the non-wetting phase. The injection rate of the wetting phase was kept constant until steady state was reached; i.e., there was no change in saturation and none of the globules were moving. The flow rate of the wetting phase was then increased stepwise until one or more non-wetting phase globule were mobilised.

During each steady-state flow condition, the evolution of the trapped non-wetting phase elements were recorded and the local velocity distributions, inside those elements, were obtained using Particle Tracking Velocimetry (PTV)^47^. Particle tracking velocimetry techniques are widely used to measure the trajectories and velocity magnitudes of moving particles and they have been successfully used in microfluidic devices coupled with fluorescent microscopy^8,47,50^. In our set-up, we used a La Vision MicroPIV system with a Carl Zeiss AxioObserver. Z1 microscope. The fluorescent particles, used as tracers of the non-wetting phase, were excited at a wavelength of 542 *nm* through a Carl Zeiss HBO 100 illuminator and identified at their emitting wavelength (612 *nm*) by a IDS CMOS camera (UI-3180CP, 2592 × 2048). The trajectory and velocity magnitude of the particles were determined from consecutive image pairs of the video sequence. The algorithm makes use of binary intensity matrices in order to determine the centroids and calculates the instantaneous velocity field from a single pair of images. Depending on the trapped non-wetting phase momentum, the frame rate of the video sequence was varied between 50 Hz to 300 Hz. The velocity vectors have been determined with an uncertainty less than 3%.

## Results and Discussions

### Introductory remarks

Our goal is to quantify and analyse the velocity distributions in trapped non-wetting phase prior and during mobilisation. For a better understanding of the process, three different types of trapped bodies are identified and investigated separately: droplets, blobs and ganglia. The trapped fluid bodies smaller than a single pore are known as droplets. Blobs are larger than a droplet and fully occupy a single pore. So, they have more contact surfaces with the solid phase. The effect of capillary forces is therefore more pronounced compared to droplets, adding complexity to the momentum transfer and mobilisation processes. Larger trapped non-wetting phase bodies, which occupy more than one pore, are referred to as ganglia. As explained above, the capillary number was increased stepwise until mobilisation of some trapped elements took place. In the cases of droplets and blobs, this covered a range of about two orders of magnitude. We focus on two key features of the process: the evolution of induced internal flow due to viscous drag and the mobilisation of the trapped phase.

### Droplets

**Figure 3:**
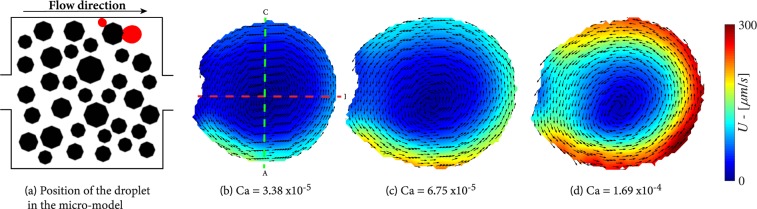
Flow distributions in a trapped droplet at various capillary numbers.

In Fig. 3, two small droplets, as two red dots, are indicated. The velocity distribution in the larger droplet at various capillary numbers (*Ca*) is also shown in Fig. 3. For this droplet, contrary to a droplet in a fluid body (see Fig. 1), two counter-rotating vortices are not observed. Instead, we see an anti-clockwise circulation cause by the local flow pattern of the wetting phase. This is because the wetting phase flows only along the underside of the droplet; there is a stagnant zone in the upper part of the droplet, as the upstream pore is blocked by the smaller droplet. Therefore, there is an asymmetry in the local velocity distribution and the centre of rotation is closer to the lower part of the droplet. In addition, lower velocities are observed at the solid-liquid interface due to the no-slip condition there. At small capillary number, the flow within the droplet is in a small region along the perimeter. The increase of capillary number leads to an inwards evolution of the flow circulations and eventually the full circulation of the non-wetting phase inside the droplet.

**Figure 4:**
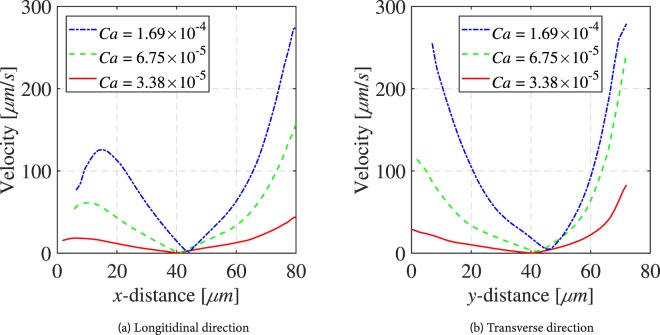
Velocity magnitudes along the red and green dashed lines in Fig. 3, respectively.

The magnitudes of velocity vectors inside the droplet along the dashed red and green lines, shown in Fig. 3, are presented in Fig. 4. As we can see, when Ca increased from 3.38 × 10^−5^ to 1.69 × 10^−4^, the wetting-phase velocity also increased reaching an average value of 4160 *μm*/*s*. Obviously, this affected the rotation intensity and the shape of the droplet. The flow velocity inside the droplet increased significantly reaching velocities of 275 *μm*/*s* at the lower side of the droplet, as shown in Fig. 4. We used the data given in Fig. 4 to calculate the drag force per unit area exerted on the droplet surface at points A, B and C. The drag force per unit area is equal to −*μdv*/*ds*, calculated at *s* = 0, where *s* is the distance from the fluid-fluid interface along the dashed line. The calculated values of drag forces for different capillary numbers are given in Table 1. As we can see, at any given capillary number, the largest drag force is exerted along the lower side of the droplet (point A), where the wetting phase flow velocity is largest. The resulting drag force from the moving wetting phase is to the right, such that a anti-clockwise circulation is created within the droplet. Because of the stagnation zone in the wetting phase in the upper part of the droplet, only a small pressure difference between upper and lower part of the droplet is expected. Therefore, the wetting phase cannot dislodge the droplet even if its flow rate is drastically increased. One should note that the droplet experiences friction (and thus resistant to mobilization) from the top and bottom surfaces of the micro-model, too. As it can be seen from Fig. 3, the droplet shape changed from circular to oval when capillary number was increased. However,, even when wetting phase capillary number was increased to 6 × 10^−3^.

**Table 1:** Calculated drag force per unit area for the cases presented in Figs 3, 5 and 6.

Globule	Ca	Drag force (per unit area) (Pa)	Drag force (per unit area) (Pa)	Drag force (per unit area) (Pa)
		Interface A	Interface B	Interface C
Droplet	1.66 × 10^−4^	2.25 × 10^−2^	3.96 × 10^−3^	9 × 10^−3^
	6.75 × 10^−5^	1.8 × 10^−2^	2.25 × 10^−3^	3.36 × 10^−3^
	3.38 × 10^−5^	1.35 × 10^−3^	4.5 × 10^−4^	1.35 × 10^−3^
Blob-1	3.95 × 10^−3^	2.25 × 10^−3^	2.7 × 10^−2^	—
	1.69 × 10^−3^	1.58 × 10^−3^	2.7 × 10^−2^	—
	1.05 × 10^−3^	1.13 × 10^−3^	2.25 × 10^−2^	—
Blob-2	4.05 × 10^−4^	2.7 × 10^−2^	1.5 × 10^−2^	1.62 × 10^−2^
	1.35 × 10^−4^	1.44 × 10^−2^	9 × 10^−3^	2.52 × 10^−2^
	6.75 × 10^−5^	1.44 × 10^−2^	6 × 10^−4^	4.95 × 10^−3^

### Blobs

**Figure 5:**
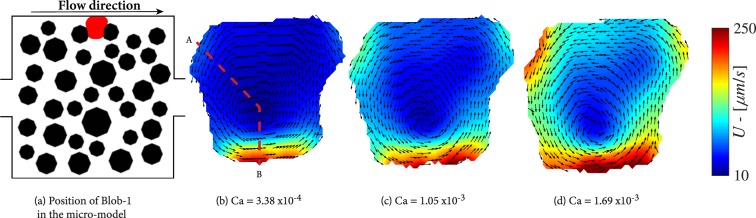
Flow distributions in a trapped blob (Blob-1) for various capillary numbers.

Figure 5 shows a blob (Blob-1) trapped between two pillars and the side wall of the micro-model; it has three liquid-liquid and three solid-liquid interfaces, as well as the liquid-solid contact area with the top and bottom surfaces of the micro-model. The velocity vectors indicate an anti-clockwise rotation of the entrapped body, which is a direct consequence of the position of the blob in the velocity field of the wetting phase and drag force(s) exerted by the wetting phase. The magnitudes of velocity vectors inside the blob along the dashed red line, shown in Fig. 5, are presented in Fig. 7. Higher velocities are observed on the lower liquid-liquid interface, where the wetting phase has its highest velocity, in comparison with the other interfaces of the blob. By increasing the capillary number from 3.38 × 10^−4^ to 1.69 × 10^−3^, the circulation velocities of the blob increase up to 300 *μm*/*s*. In addition, the rotation centre is shifted towards the high velocity region approaching the lower liquid-liquid interface. Note also that for all capillary numbers, higher velocities are reached at the liquid-liquid interfaces while the internal blob velocities at the solid-liquid boundaries remain small. The above trends are more pronounced for the larger capillary number of 1.69 × 10^−3^. The drag forces at points A and B are calculated and presented in Table 1. It is clear that the drag force at the downstream interface is much larger and is responsible for anti-clockwise direction of rotation.

**Figure 6:**
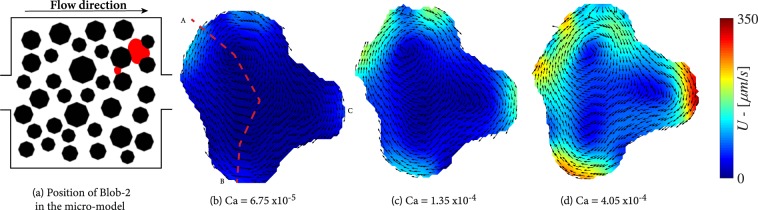
Flow distributions in a trapped blob (Blob-2) for various capillary numbers. Note that in this experiment, the micro-model was rotated 180°.

**Figure 7:**
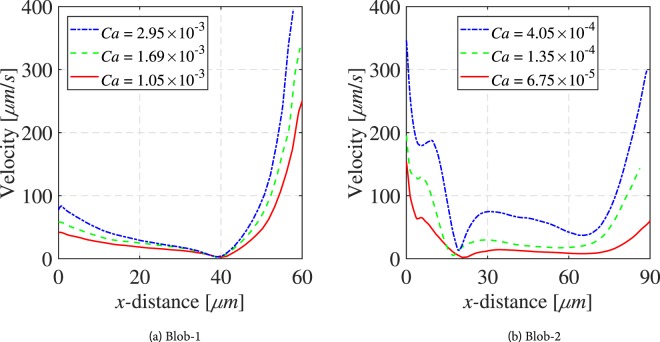
Velocity magnitudes inside Blob-1 and Blob-2 along the red dashed lines shown in Figs 5 and 6, respectively.

Figure 6 shows another experiment with an entrapped blob (Blob-2) having a fidget spinner-like shape and with its arms being the liquid-liquid interfaces. Here, the position of the blob in the wetting velocity field is such that the circulation direction in the blob is clockwise. This is due to the fact in this case the wetting phase flows over the upper and right sides of the blob; a small droplet prevents the wetting fluid from flowing below the blob. This can be clearly observed as the capillary number increases from 6.75 × 10^−5^ to 4.05 × 10^−4^. The maximum velocities are observed at the downstream interface reaching values of 350 *μm*/*s* while the momentum transfer at the bottom interfaces is significantly lower. This is all evident from the plot of magnitudes of velocity vectors inside the blob along the dashed red line presented in Fig. 7. The drag forces per unit area at points A, B and C (shown in Fig. 6) are calculated and presented in Table 1. It is clear that the drag force along interface B is larger and together with the drag force along interface A cause the clockwise direction of rotation in the blob.

First, despite their similar size, for the same capillary numbers, Blob-2 shows higher circulation velocities than Blob-1, as shown in Fig. 6. The is due to the difference in active liquid-liquid interfacial area, across which momentum transfer occurs. The ratio of liquid-liquid interfacial area–to–total interfacial area (*S*_*ll*_/*S*_*tot*_) for the two blobs is fairly similar; 22.24% and 23.37%, respectively (see Table 2). In case of Blob-1, the drag force is applied only to the lower interface, with an area of about 5000 *μ*m^2^. Whereas, in the case of Blob-2, the drag force acts on the most upstream and the most downstream interfaces, with a total area of 9700 *μ*m^2^. Also, the location at which a blob is trapped is important. Higher wetting phase velocities are expected near the centre of the micro-model and Blob-2 is closer to this region.

**Table 2:** Geometrical characteristics of the non-wetting phase clusters.

	S_tot_ (μm^2^)	S_sl_(μm^2^)	S_ll_(μm^2^)	Ss_l_/S_tot_	S_ll_/S_tot_
Droplet	16800	2250	14500	13,3%	86,6%
Blob-1	52336	40716	11650	77.76%	22.24%
Blob-2	55430	42480	12950	76.63%	23.37%
Ganglion	62800	31850	30950	50.07%	49.93%

**Figure 8:**
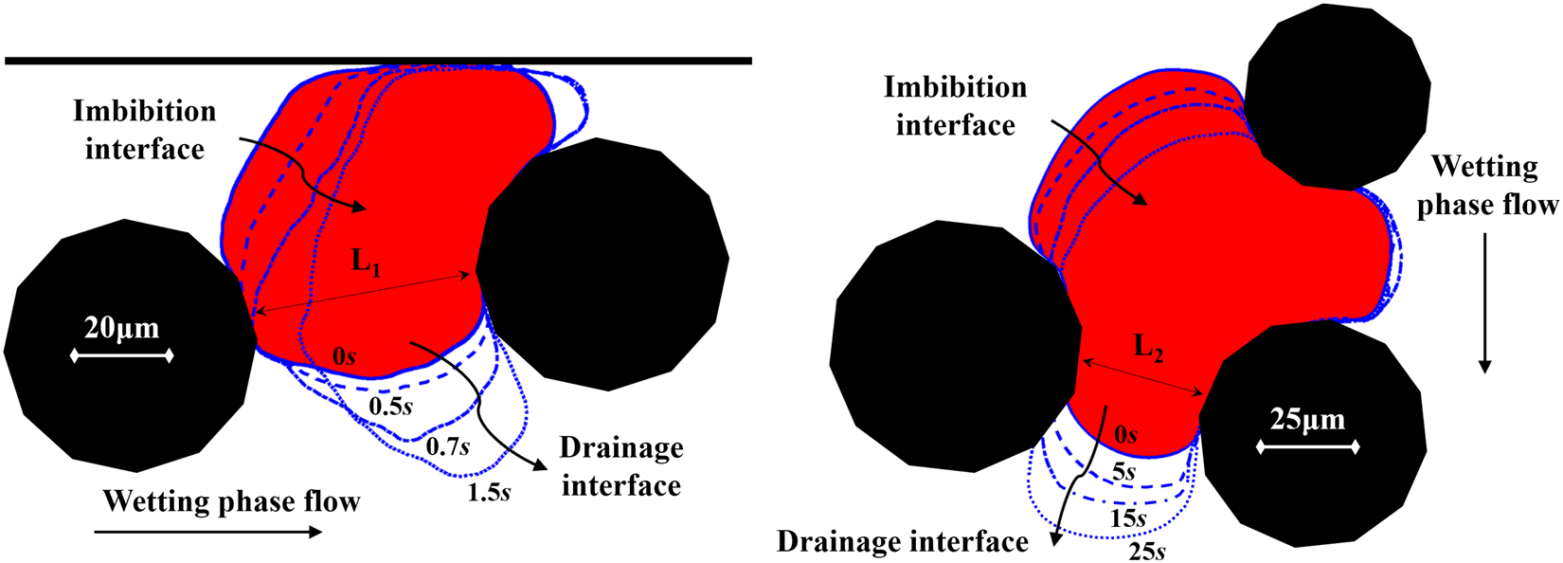
Instantaneous mobilisation contours for (**a**) Blob-1 at *Ca* = 6.75 × 10^−3^ and (**b**) Blob-2 at *Ca* = 5.4 × 10^−4^.

The mobilisation of blobs is a process that is clearly linked to the momentum transfer from the wetting phase. However, the pore structure and the behaviour of other trapped clusters also play an important role and add to the complexity of the process. First, we note that when there is flow and at any capillary number, the upstream interface is rather flat under the pressure of the non-wetting phase. The downstream interface, however, has a larger curvature and thus a higher capillary pressure so that the downstream pressure within the non-wetting phase is large. This ensures that, although there is a pressure gradient within the wetting phase, the effective pressure difference within the non-wetting phase remains zero; so that the non-wetting phase does not flow. There would be a small pressure gradient induced by the flow circulation within the globule. At larger capillary numbers, the upstream interface becomes slightly flatter and downstream interface bulges more (see the three images in Figs 5 and 6), in order to accommodate the larger pressure gradient within the wetting phase and the globule remains trapped. This change of curvatures of upstream and downstream interfaces cannot be sustained as the wetting phase flow rate is increased and, at some capillary number, mobilization would occur. The mobilisation of Blob-1 took place at a Ca 20 times larger than the Ca at which it was trapped. For the case of Blob-2, the required Ca was 8 times larger. At the mobilization capillary number, one of the interfaces become unstable, a pressure gradient is established within the globule and it starts to flow. Figure 8 shows instantaneous mobilisation snapshots at the critical capillary number. Note that velocity vectors were not possible to be recorded during mobilisation due to the limited frame grabbing capabilities with reasonable resolution. However, the tracing of the fluorescent particles allowed a real-time visualisation experiment and the various outlines of the blobs during the mobilisation process are extracted and shown in Fig. 8. While Blob-1 is mobilised fully and starts to leave the pore within 1.5 seconds, it takes Blob-2 more than 25 seconds to get mobilised. The reason for this large difference in mobilization time is the size of the downstream pore throat that get invaded. For Blob-1, the pore throat size L1 is much larger than L2 for Blob-2. This means that the resistance offered to the flow of the ganglion is much less for Blob-1 than for Blob-2 and it takes less time to leave the pore. Also, as Blob-1 starts to move, the pore opens up to the flowing wetting phase (at 0.7 sec after mobilization; see Fig. 8a) and wetting phase exerts significant drag force on the globule and accelerates the full mobilization. This does not happen in the case of Blob-2.

### Ganglia

**Figure 9:**
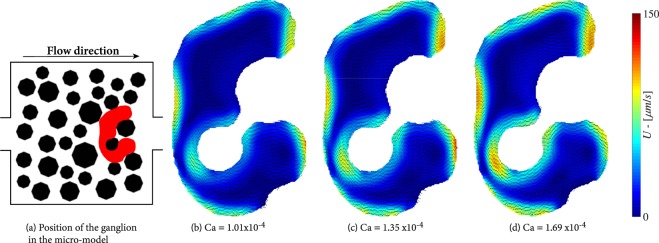
Flow distributions in a trapped ganglion for various capillary numbers.

Figure 9 shows a ganglion that occupies five pore bodies and has eight solid-liquid and eight liquid-liquid interfaces. This ganglion was formed under a capillary number of 1.01 × 10^−4^. The wetting fluid flows mostly downwards along the upstream interface of the ganglion and creates four vortices, all rotating anti-clockwise and forming a loop-pulley flow pattern. The rotation direction is controlled by the location of the ganglion in the micro-model and the wetting phase flow direction and velocity.

**Figure 10:**
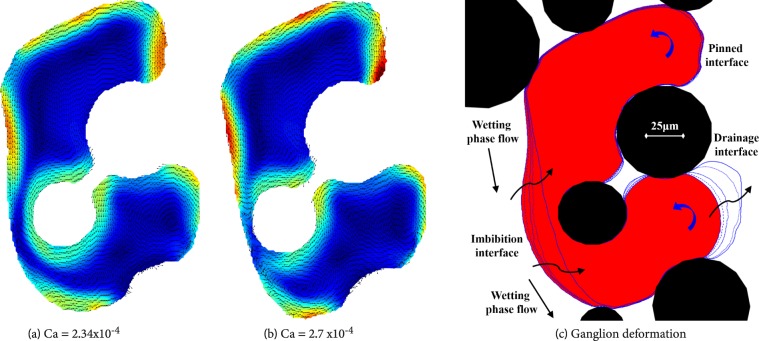
Flow distributions in a ganglion during deformation, prior to break-up.

As in the case of blobs, flow velocity is highest along the liquid-liquid interfaces. However, there is an area of relatively high velocity near the solid-fluid interface that is in the inner side of the ganglion. The wetting phase capillary number was increased stepwise, with a constant increment of 0.34 × 10^−4^, up to Ca = 2.7 × 10^−4^, at which the ganglion was broken up. The flow fields within the ganglion at different capillary numbers are shown in Figs 9 and 10. We see a significant deformation of the ganglion, with the upstream interface flattening under the pressure of the wetting phase and being pushed towards the solid-liquid interface. At the same time, the curvature of the most downstream interface increases. This is made evident in Fig. 10c, where contour lines of the ganglion at different capillary numbers are drawn. We see the thinning of a filament of non-wetting phase formed next to the inner solid-liquid interface. At the highest capillary number, the downstream interface becomes unstable, flow within the thin filament of non-wetting phase virtually stops and the ganglion ruptures there.

Breakup of a ganglion is a common occurrence that leads to a partial mobilisation of ganglia. The breakup, as described by Lenormand & Sarr^12^, is commonly characterised by the deformation of the ganglion and the formation of a non-wetting phase filament. The increase of wetting phase flow local velocity and pressure causes the filament to get thinner and eventually to rupture. Due to the rapture, two daughter ganglia are formed. This happened in the case of the ganglion shown in Figs 9 and 10.

**Figure 11:**
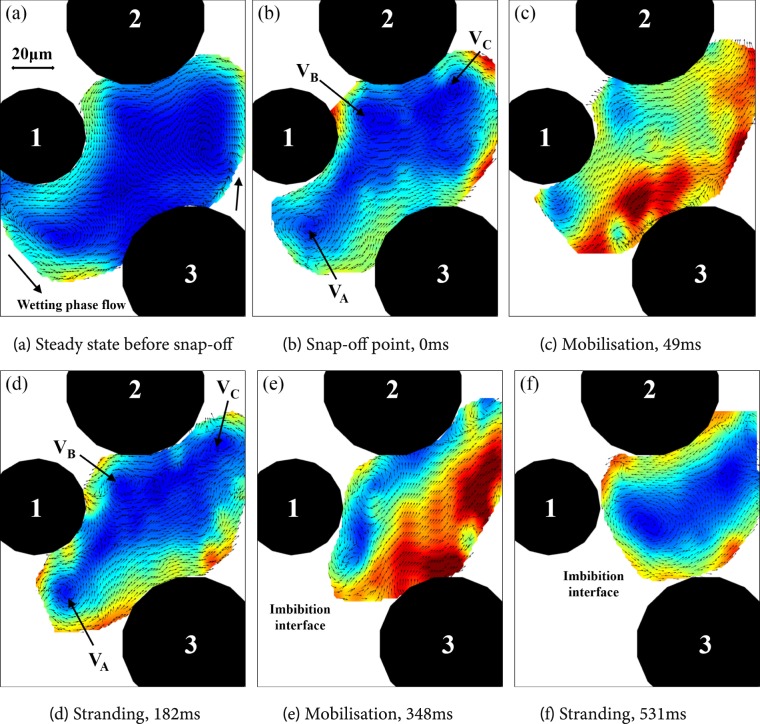
Instantaneous velocity vectors during snap-off and mobilisation of the ganglion.

After breakup, the upper part of the ganglion remained stranded while the lower part was mobilised and moved as a blob. Its locations at different times at the time of breakup and after that are shown in Fig. 11. The mobilised lower part moved only for a short distance and time and it got stranded in the downstream pore. Clearly, the capillary number and associated pressure gradient in the wetting phase was not large enough to keep it mobilised. The movement of the blob during the mobilization phase was a combination of very brief pause (Fig. 11d) and further mobilization. Immediately after breakup, three vortices were formed (indicated by VA, VB and VC in Fig. 11b). The vortices disappeared as the blob moved further (Fig. 11c), reappeared again as the blob was briefly stranded (Fig. 11d), disappeared as the blob moved on (Fig. 11e) and finally two vortices were established after stranding (Fig. 11f). When the globule moves, the flow velocities almost everywhere within the globule are approximately equal and in the direction of its movement.

## Conclusions

We performed two-phase flow experiments with focus on observing the evolution of flow within trapped non-wetting phase globules prior, during and after their mobilisation. Flow visualisation and quantitative velocity measurements were obtained using Particle Tracking Velocimetry (PTV).

The experimental results show the interplay of capillarity and momentum transfer between the two fluid phases for different trapped globules. At all time, when the non-wetting phase globules (droplets, blobs and ganglia) are trapped, vortices are formed within them. The circulation velocities are larger at the fluid-fluid interfaces, along which the wetting phase flows and decreases towards the middle of the globule, which is a stagnation zone. In the case of ganglia (i.e., large globules), more than one vortex are formed. The circulation velocities increased proportionally with the increase of wetting phase average velocity (or capillary number). For example, when the average wetting phase average velocity increased from 16.670 *μm*/*s* to 40.000 *μm*/*s*, the maximum velocity within a blob increased up to 350 *μm*/*s*. The vortices remain stable as long as the globules are trapped and start to change at the onset of mobilization and disappear during the movement of the globules. They reappear when the globules get stranded, again.

The trapped globules can become mobilised due to the momentum transfer from the wetting phase; this occurs in the form of viscous drag as well as a difference in the local wetting phase pressure between upstream and downstream liquid-liquid interfaces of the globules. Droplets are trapped at a relatively low capillary number and do not mobilise even at high capillary numbers. This is because they can adopt their shape in order to minimise the drag force. Also, the pressure difference in the wetting phase along the droplet is not large enough to dislodge it. Blobs can get mobilised once the momentum transfer is large enough to dislodge them. In concordance with the literature, Blob-1 was mobilised at a Ca 20 times larger than the trapping Ca^5^. However, Blob-2 was mobilised at Ca 8 times larger than the trapping Ca. This concludes that the local flow conditions are the main controlling factors for their mobilisation. A blob trapped near the side walls of the micro-model was less prone to mobilisation than those trapped more towards the middle of the micro-model.

The flow field within a ganglion is much more complex than the other two globule types. There could be circulation velocities near liquid-solid interfaces and in some regions the circulation flow may stop when capillary number increases. At sufficiently high capillary number, as the drag force and the pressure of wetting phase on various interfaces of a ganglion increase, the ganglion gets ruptured at the area where there is no flow within the ganglion and parts of it is mobilised. This typically happens at a lower capillary number than the critical capillary number of blobs. Therefore, the mobilised part becomes stranded again.

Our results show the evolution of the internal flow within non-wetting phase trapped globules and the mobilisation of discontinuous phases. For obtaining more information about the momentum exchange between phases, it would be desirable to dope the wetting phase with fluorescent particles and perform direct visualisation of both flow fields.
